# Configurationally stable, enantioenriched organometallic nucleophiles in stereospecific Pd-catalyzed cross-coupling reactions: an alternative approach to asymmetric synthesis

**DOI:** 10.1039/c5sc01710f

**Published:** 2015-07-01

**Authors:** Chao-Yuan Wang, Joseph Derosa, Mark R. Biscoe

**Affiliations:** a Department of Chemistry , The City College of New York (CCNY) , 160 Convent Avenue , New York NY 10031 , USA . Email: mbiscoe@ccny.cuny.edu; b The Graduate Center of the City University of New York (CUNY) , 365 Fifth Avenue , New York NY 10016 , USA

## Abstract

Several research groups have recently developed methods to employ configurationally stable, enantioenriched organometallic nucleophiles in stereospecific Pd-catalyzed cross-coupling reactions. By establishing the absolute configuration of a chiral alkyltin or alkylboron nucleophile prior to its use in cross-coupling reactions, new stereogenic centers may be rapidly and reliably generated with preservation of the known initial stereochemistry. While this area of research is still in its infancy, such stereospecific cross-coupling reactions may emerge as simple, general methods to access diverse, optically active products from common enantioenriched organometallic building blocks. This minireview highlights recent progress towards the development of general, stereospecific Pd-catalyzed cross-coupling reactions using configurationally stable organometallic nucleophiles.

## Abstract 2

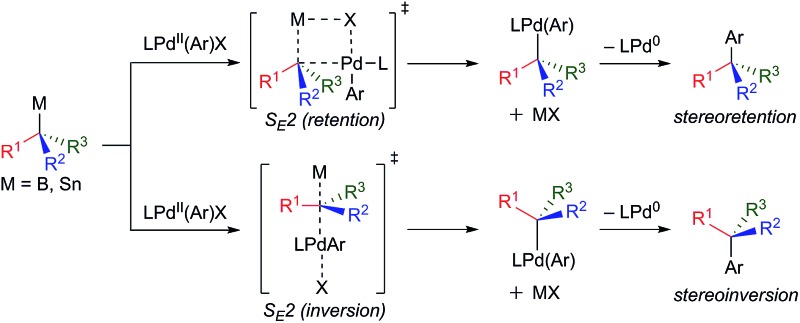
Several research groups have recently developed methods to employ configurationally stable, enantioenriched organometallic nucleophiles in stereospecific Pd-catalyzed cross-coupling reactions.

## Introduction

Asymmetric catalysis is commonly employed in the creation of stereogenic centers during carbon–carbon bond construction.^1,2^ However, it is typically difficult to predict the effect of small steric and/or electronic perturbations of the substrate on the level of asymmetric induction transferred to the product. When asymmetric processes are applied broadly in diversity oriented synthesis, a small modification of a specific architectural motif often results in significantly eroded enantioselectivities. Extensive re-optimization of reaction conditions, or the use of different chiral catalysts is often required to improve asymmetric induction. This lack of generality greatly complicates attempts to employ asymmetric catalysts rationally and predictably. Recently, multiple research groups have reported the use of optically active alkyltin^3^ and alkylboron^4^ nucleophiles in stereospecific Pd-catalyzed methods to generate enantioenriched products.^5,6^ In these reactions, the stereocenter is preformed on a configurationally stable, enantioenriched main group organometallic reagent. If the stereochemical integrity of the nucleophile could be preserved throughout the C–C bond-forming reaction, these reactions would enable the development of general cross-coupling reactions with stereospecificity that is independent of electronic and steric perturbations of the coupling partners. In this minireview, we present recent progress towards the development of general, stereospecific Pd-catalyzed cross-coupling reactions using configurationally stable organometallic nucleophiles.

**Fig. 1:**
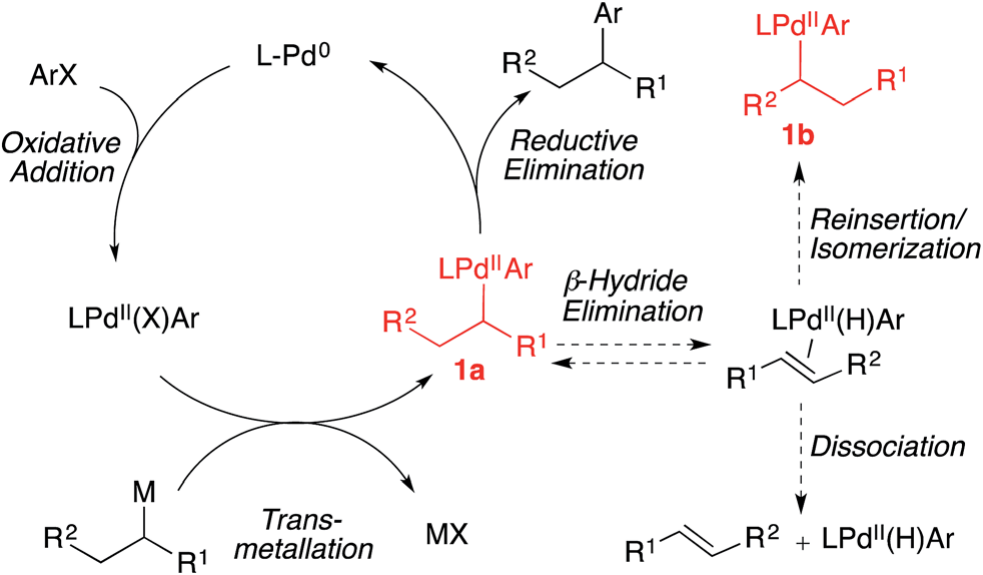
Catalytic cycle and competing processes for Pd-catalyzed cross-coupling reactions of secondary nucleophiles and aryl electrophiles.

**Fig. 2:**
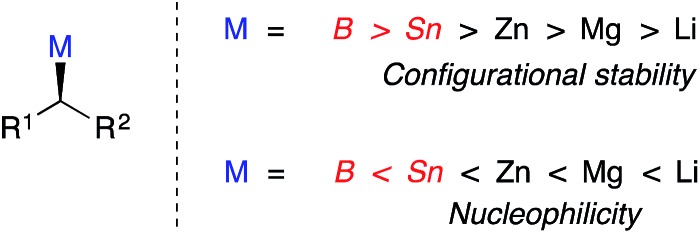
Inverse relationship between configurational stability and nucleophilicity for main group organometallic nucleophiles.

Over recent decades, palladium-catalyzed C(sp^2^)–C(sp^2^) cross-coupling reactions have become reliable, routine, high-yielding processes.^7^ More recently, methods for achieving C(sp^2^)–C(sp^3^) and C(sp^3^)–C(sp^3^) cross-coupling reactions have been investigated.^8^ Conceptually, a stereogenic center can be generated *via* a stereospecific C–C bond-forming cross-coupling reaction involving an optically active secondary or tertiary alkyl main group organometallic nucleophile. However, the use of alkyl organometallic nucleophiles in metal-catalyzed cross-coupling reactions is particularly challenging due to the propensity of the alkyl ligand of intermediate **1a** to undergo β-hydride elimination (Fig. 1).^7,8^ After β-hydride elimination, reductive elimination leads to a reduced aryl product alongside an olefin product. Reinsertion of the palladium hydride into the coordinated olefin can result in the formation of a racemic product and/or isomerization to a new branched (secondary/tertiary) (**1b**) or linear (primary) group, depending on the structure of the secondary nucleophile. The development of general strategies to employ configurationally stable organometallic nucleophiles in cross-coupling reactions is additionally impeded by the inverse relationship that exists between the nucleophilicity and configurational stability of carbon–metal bonds in main group organometallic nucleophiles.^9^ While increased covalency tends to coincide with enhanced configurational stability of the carbon–metal bond, it also tends to coincide with reduced nucleophilicity (Fig. 2). This trend, in addition to the inherent steric bulk of secondary and tertiary alkyl nucleophiles, can result in prohibitively slow transmetallation of such nucleophiles as the covalency of the carbon–metal bond increases.

Alkyltin and alkylboron reagents exhibit particularly high configurational stability, and constitute the most viable nucleophiles for broad use in stereospecific cross-coupling processes.^9^ Many optically active alkyltin and alkylboron compounds are isolable and configurationally stable under ambient conditions. Prior to recent efforts, stereospecific Pd-catalyzed cross-coupling reactions of C(sp^3^) nucleophiles typically required the use of cyclopropyl reagents.^10^ However, because cyclopropyl groups undergo uniquely facile transmetallation and cannot undergo β-hydride elimination, such reactions constitute markedly limited examples of stereospecificity in cross-coupling reactions. By comparison, an efficient, general method to employ optically active alkyltin and alkylboron nucleophiles in palladium-catalyzed cross-coupling reactions would constitute a broadly powerful tool for use in organic synthesis.

**Fig. 3:**
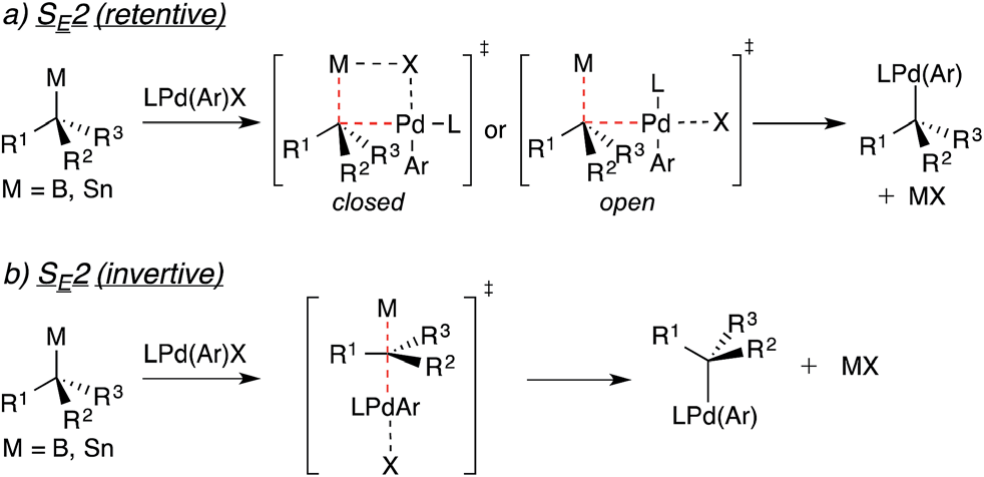
Potential mechanisms for the stereospecific transmetallation of optically active alkyltin and alkylboron nucleophiles to palladium.

The stereospecificity of Pd-catalyzed cross-coupling reactions involving enantiomerically enriched nucleophiles is determined by the mechanism through which transmetallation occurs.^11,4e^ Transmetallation *via* a closed or open S_E_2 mechanism as depicted in Fig. 3a will result in enantioretention. Transmetallation *via* an S_E_2 mechanism utilizing the minor bonding lobe of the C–B/Sn bond (Fig. 3b) will result in enantioinversion. Involvement of radical pathways will lead to stereochemical erosion or racemization. In light of the multiple pathways by which the transmetallation of an alkyl units may occur, a strong mechanistic underpinning for transmetallation is necessary for such reactions to be employed predictably. In Suzuki cross-coupling reactions, previous mechanistic studies strongly suggest that formation of ArPd(OH)L (*i.e.*, X = OH) is required for transmetallation of arylboron nucleophiles.^12^ It is likely that the ArPd(OH)L complex is similarly involved in the transmetallation of alkylboron nucleophiles.

## Use of enantioenriched secondary alkyltin nucleophiles

The utility of alkylstannane reagents in cross-coupling reactions is complicated by the requirement for four substituents on the tin center. This problem is circumvented in traditional Stille reactions by exploiting the enhanced migratory aptitude of C(sp) and C(sp^2^) substituents relative to C(sp^3^) substituents on tin.^7^ Three alkyl substituents can be employed as inert “dummy ligands” in cases where the selective transfer of an alkynyl, alkenyl, or aryl substituent is desired. For tetraalkylstannane nucleophiles, transfer of only one alkyl unit is typically observed; three potentially precious alkyl units are sacrificed during this process. Thus, the use of tetraalkylstannane nucleophiles is limited by our ability to effect the selective transfer of one alkyl substituent of a tetraalkylstannane bearing three expendable alkyl units. It has been demonstrated that the presence of a C(sp^2^) α-carbon, an α-heteroatom, and/or a coordinating group can facilitate selective alkyl transfer from a tetraalkylstannane.^3^ Most examples of stereospecific Stille cross-coupling reactions require such activation of the secondary alkylstannane reagent to promote selective transmetallation.

**Fig. 4:**
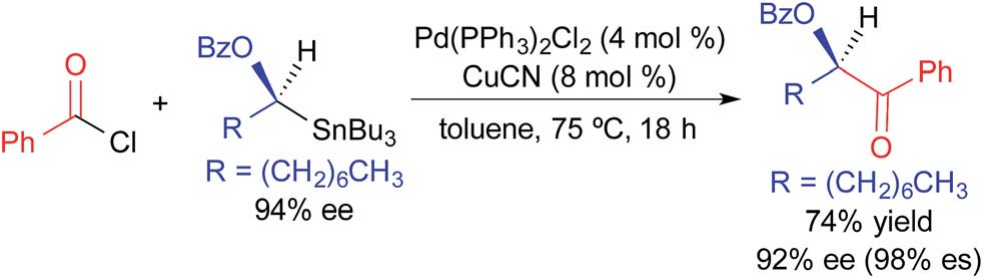
Stereospecific Pd-catalyzed cross-coupling reaction of an activated, enantioenriched alkylstannane and benzoyl chloride as reported by Falck.

In 1994, Falck exploited the enhanced propensity for transmetallation of an enantioenriched alkyl unit bearing an α-OBn group in a Pd-catalyzed acylation reaction (Fig. 4).^3*a*^ This reaction was highly enantiospecific,^13^ occurring with retention of absolute configuration. Since copper(i) cyanide was employed as a co-transmetallating agent, this work suggests that transmetallation from tin to copper, and from copper to palladium, occurs stereospecifically. While only one example was provided in this study, it established an important precedent for the use of activated tetraalkylstannanes in Pd-catalyzed cross-coupling reactions.

**Fig. 5:**
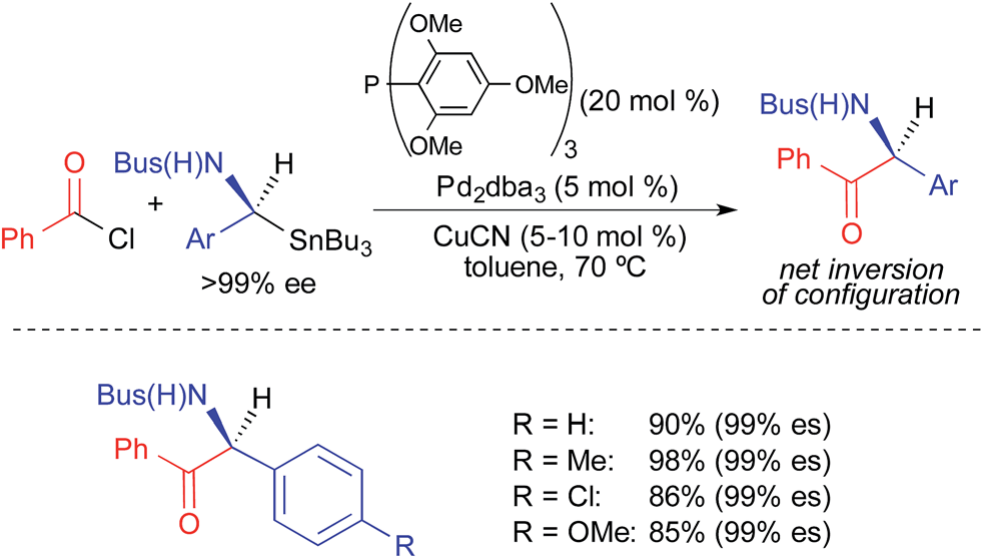
Stereospecific Pd-catalyzed cross-coupling reactions of activated, enantioenriched benzylstannanes and benzoyl chloride as reported by Chong.

Chong extended the use of α-heteroatomic stannanes to highly stereospecific cross-coupling reactions of enantioenriched benzylic α-sulfonamidostannanes and benzoyl chloride (Fig. 5).^3*b*^ In contrast to the Falck study, inversion of absolute configuration was reported in these reactions. This work illustrates the unpredictable influence that structural modifications of the stannane nucleophile could have on the mechanism of transmetallation.^14^

**Fig. 6:**
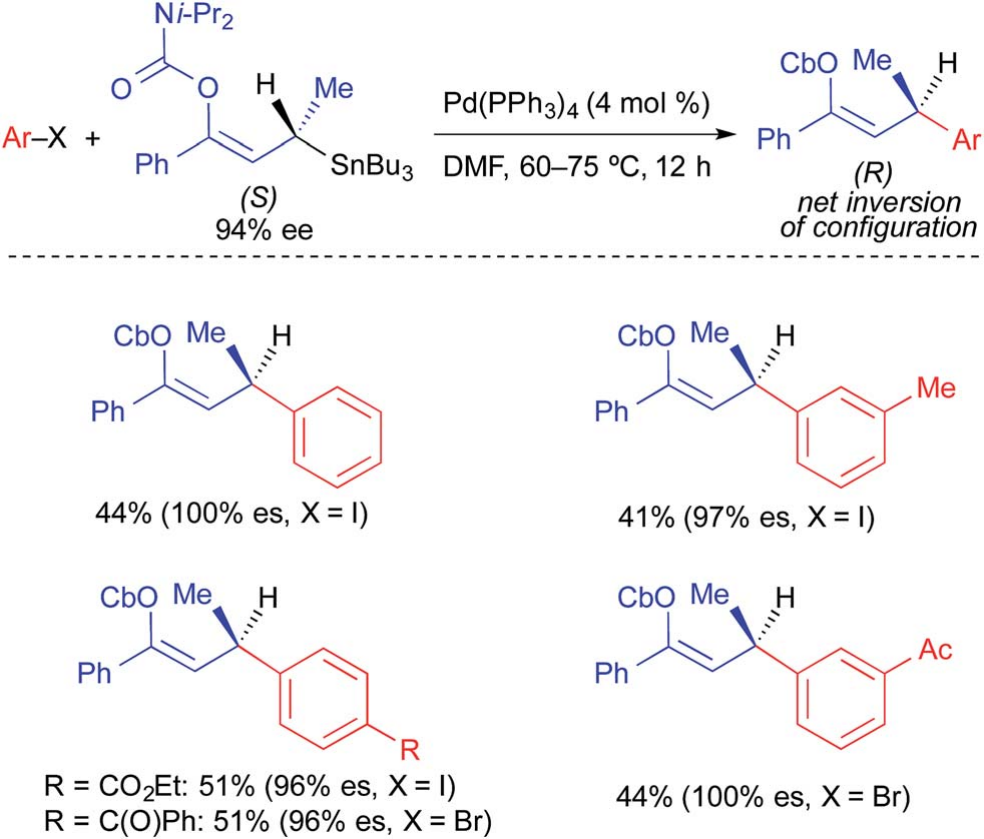
Stereospecific Pd-catalyzed cross-coupling reactions of activated, enantioenriched allylic stannanes and aryl electrophiles as reported by Hoppe.

In 2006, Hoppe reported the first example of stereospecific transfer of an activated C(sp^3^) unit from an organotin nucleophile in a Pd-catalyzed arylation reaction (Fig. 6).^3*c*^ In this reaction, an allylic stannane bearing an alkenyl carbamate was employed as a nucleophile in cross-coupling reactions with simple aryl iodides and bromides. Similar to the Pd-catalyzed cross-coupling reactions reported by Chong, this reaction proceeded with net inversion of absolute configuration. While this process showed high stereospecificity, biproducts derived from allylic transposition of the carbamate were typically observed alongside the desired cross-coupling product.

**Fig. 7:**
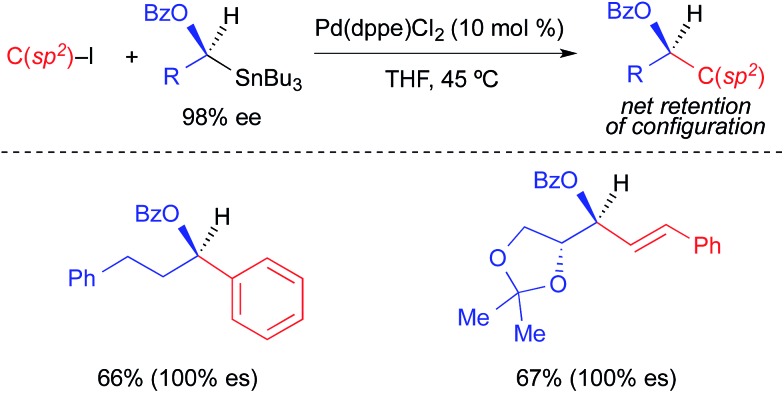
Stereospecific Pd-catalyzed cross-coupling reactions of activated, enantioenriched alkylstannanes and aryl/alkenyl iodides as reported by Falck.

Falck extended the use of α-heteroatom-activated alkylstannanes to reactions involving aryl and vinyl halides (Fig. 7).^3*d*^ Consistent with the original report using benzoyl chloride (Fig. 4), these reactions proceeded stereospecifically with net retention of absolute configuration. While only two examples of Pd-catalyzed cross-coupling reactions using optically active alkylstannanes and aryl/vinyl halides were provided, multiple racemic examples were demonstrated using different vinyl, aryl, and heteroaryl electrophiles. This suggests a reasonable likelihood that these reactions are general with respect to the structure of the electrophilic coupling partner.

**Fig. 8:**
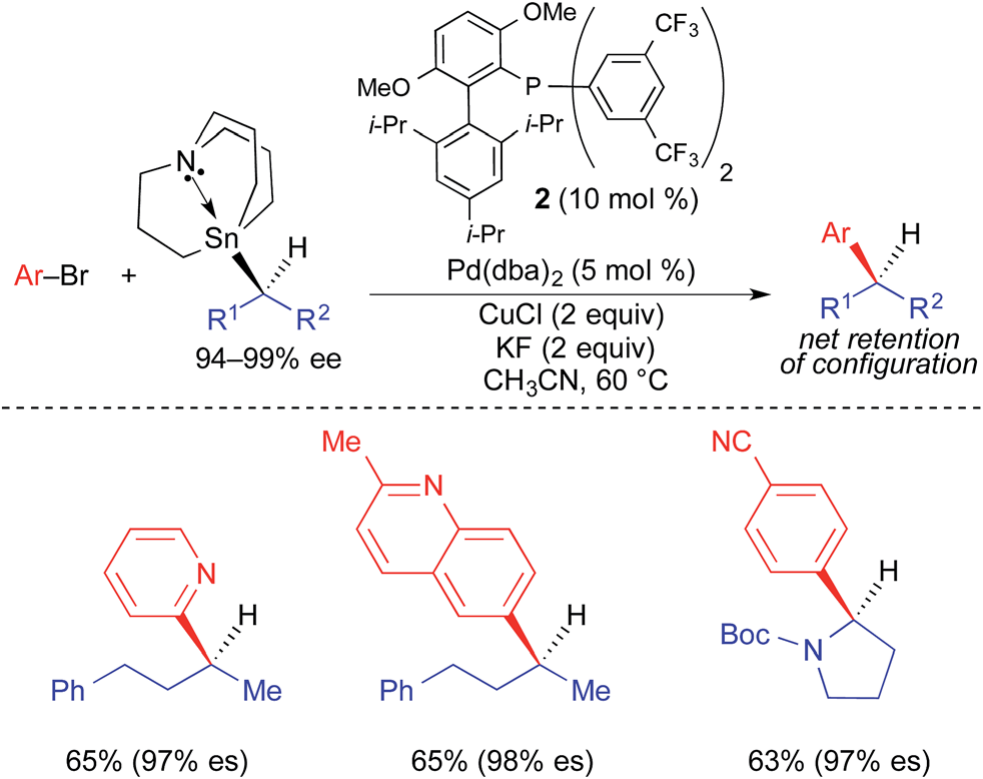
Stereospecific Pd-catalyzed cross-coupling reactions of unactivated, enantioenriched secondary alkylcarbastannatranes and aryl bromides as reported by Biscoe.

In 2013, our research group reported the first example of a stereospecific Pd-catalyzed cross-coupling reaction using an unactivated, enantioenriched nucleophile (Fig. 8).^3*e*^ In this work, selective alkyl transfer from a tetraalkylstannane was achieved using an alkylcarbastannatrane^15^ nucleophile. Jurkschat and Tzschach showed that the nitrogen atom in the atrane backbone selectively lengthens the Sn–C bond of the apical alkyl substituent by 0.1 Å compared to the Sn–C bond of a tetraalkylstannane.^16^ Vedejs subsequently exploited the increased lability of the apical alkyl group of a carbastannatrane, achieving selective transfer of a primary alkyl group in Pd-catalyzed Stille reactions.^17^ Our group extended this work to the use of secondary alkylcarbastannatranes, which resulted in the development of a highly general process for Pd-catalyzed cross-coupling reactions of secondary alkyl nucleophiles and aryl/heteroaryl electrophiles. This reaction required the use of Cu(i) as a co-transmetallating reagent in order to achieve efficient transfer of the secondary alkyl unit to palladium.^18^ JackiePhos (**2**),^19^ a bulky, electron-deficient biarylphosphine ligand, was uniquely effective at supporting this process. Using these conditions, unactivated, configurationally stable, optically active alkylcarbastannatrane nucleophiles could be employed in highly stereospecific cross-coupling reactions (Fig. 8). These reactions proceeded with retention of absolute configuration. Considering the generality of the corresponding racemic process, it is likely that the stereospecific variant is limited only by the current lack of versatile methods by which to produce optically active alkylcarbastannatrane reagents.

**Fig. 9:**
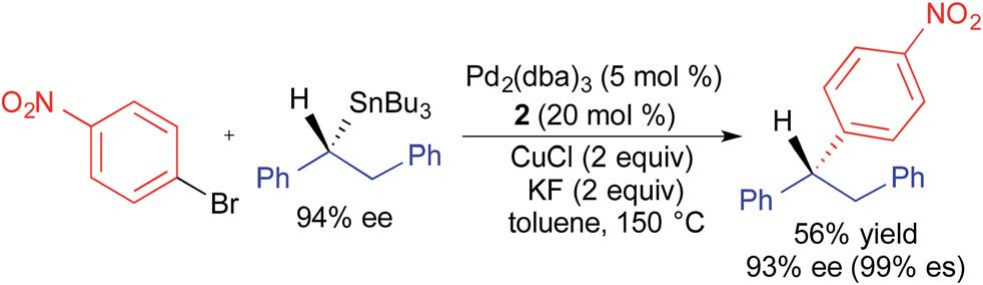
Stereospecific Pd-catalyzed cross-coupling reaction of an enantioenriched benzylstannane and an aryl bromide as reported by Liao.

Recently, Liao employed similar reaction conditions to those in Fig. 8 to achieve the highly stereospecific coupling of an enantiomerically enriched benzylic tributylstannane and an aryl bromide (Fig. 9).^3*f*^ The enhanced migratory aptitude of the benzyl fragment was exploited to achieve selective transfer without the use of a carbastannatrane reagent. Since the one reported example involved the use of an activated (*i.e.*, electron-deficient) aryl bromide, it is unclear how general this process is with respect to electrophile scope. Additionally, the absolute stereochemistry of the product was inferred through analogy to our stereoretentive alkylcarbastannatrane work, and not rigorously assigned. While the stereochemical assignment is likely correct, we recommend caution when proposing absolute stereochemistry based upon precedents that were obtained using nucleophiles with different modes of activation.

## Use of enantioenriched secondary alkylboron nucleophiles

Unlike alkylstannane nucleophiles, alkylboron nucleophiles generally contain only one alkyl unit. Therefore, alkylboron nucleophiles do not require selective alkyl transfer in Pd-catalyzed cross-coupling reactions. However, the increased covalency of the carbon–boron bond results in the reduced nucleophilicity of alkylboron reagents compared to alkylstannanes. As with alkylstannanes, the presence of a C(sp^2^) α-carbon, an α-heteroatom, and/or a coordinating group can facilitate alkyl transfer from alkylboron reagents. Indeed, most examples of stereospecific Suzuki cross-coupling reactions require such activation of the secondary alkylboron reagent to effect transmetallation and/or prevent β-hydride elimination following transmetallation. Because many reliable processes to generate optically active secondary alkylboron nucleophiles have been developed,^20^ a general method to employ secondary alkylboron nucleophiles in stereospecific cross-coupling reactions would have far-reaching applications.

**Fig. 10:**
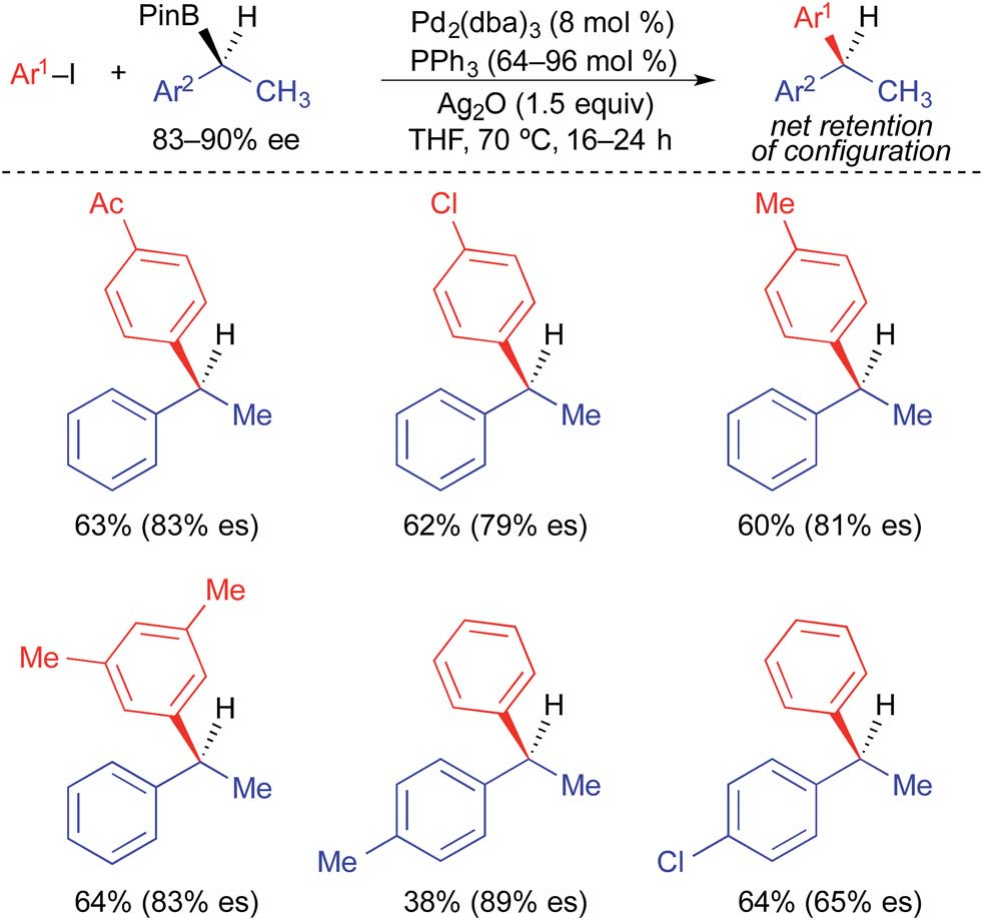
Stereospecific cross-coupling reactions of enantioenriched benzylboronic esters and alkyl iodides as reported by Crudden.

**Fig. 11:**
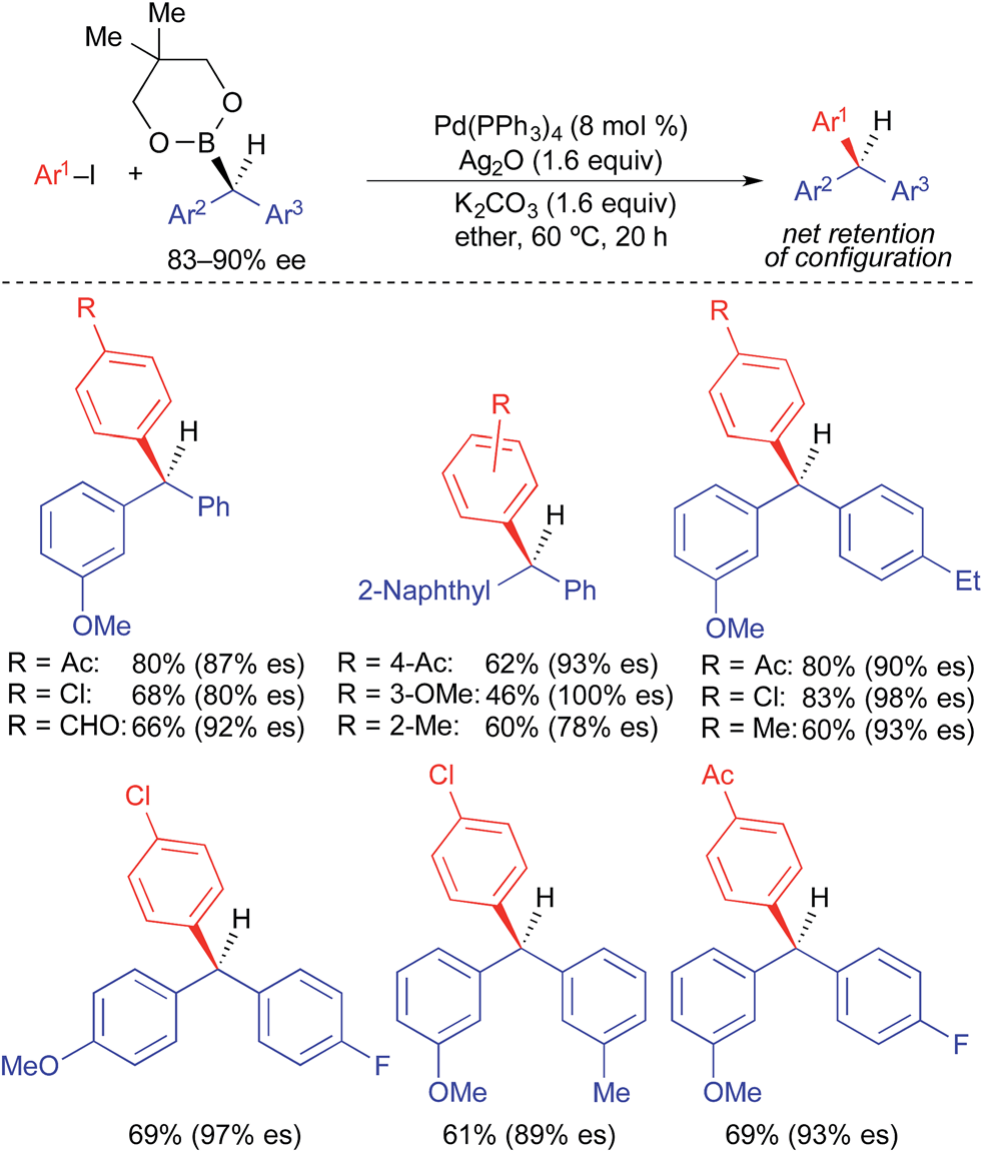
Stereospecific Pd-catalyzed cross-coupling reactions of enantioenriched dibenzylic organoboronic esters and aryl iodides as reported by Crudden.

Using optically active, benzylic organoboronic esters, Crudden demonstrated the first stereospecific examples of Pd-catalyzed Suzuki reactions between secondary alkylboron nucleophiles and aryl electrophiles (Fig. 10).^4a,21^ Stoichiometric Ag(i) was employed in these reactions to promote the formation of cationic Pd(ii) intermediates, to which facile transmetallation of the benzylic nucleophile occurred. Reactions were limited to electron-deficient and electron-neutral aryl iodides, and no heteroaromatic electrophiles were employed. Net retention of absolute stereochemistry was demonstrated in this reaction, with moderate to good levels of stereospecificity. Using modified conditions in which neopentylglycol boronic esters were employed in place of the pinacol boronic esters, Crudden recently extended the reaction to the use of optically active, dibenzylic nucleophiles (Fig. 11), which enabled the preparation of enantioenriched triarylmethanes.^4*j*^ These reactions proceeded with greater stereospecificity than the original reactions that used enantioenriched monobenzylic organoboronic esters. The substrate scope was still limited to electron-deficient and electron-neutral aryl iodides, and the coupling reaction again occurred with retention of absolute stereochemistry.

**Fig. 12:**
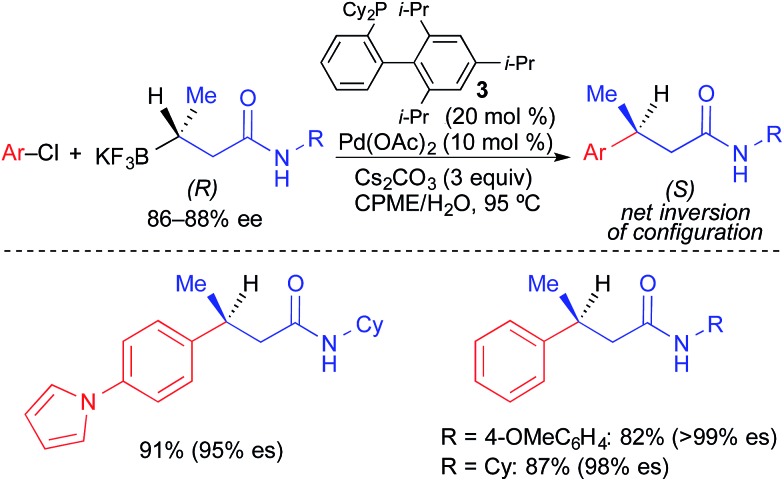
Stereospecific Pd-catalyzed cross-coupling reactions of enantioenriched alkyl β-trifluoroboratoamides and aryl chlorides as reported by Molander.

Molander reported the use of secondary alkyl β-trifluoroboratoamides in highly stereospecific Pd-catalyzed cross-coupling reactions with aryl electrophiles (Fig. 12).^4*b*^ XPhos (**3**), a bulky, electron-rich biarylphosphine ligand, was required in this process. The reaction proceeded efficiently with electron-deficient and electron-neutral aryl bromides and chlorides. Unlike the Suzuki couplings reported by Crudden in which transmetallation occurred primarily with stereoretention, transmetallation in this reaction proceeded predominantly with stereoinversion. It was proposed that intramolecular coordination of the amide to palladium promotes invertive transmetallation of the alkyltrifluoroborate, while also retarding β-hydride elimination.

**Fig. 13:**
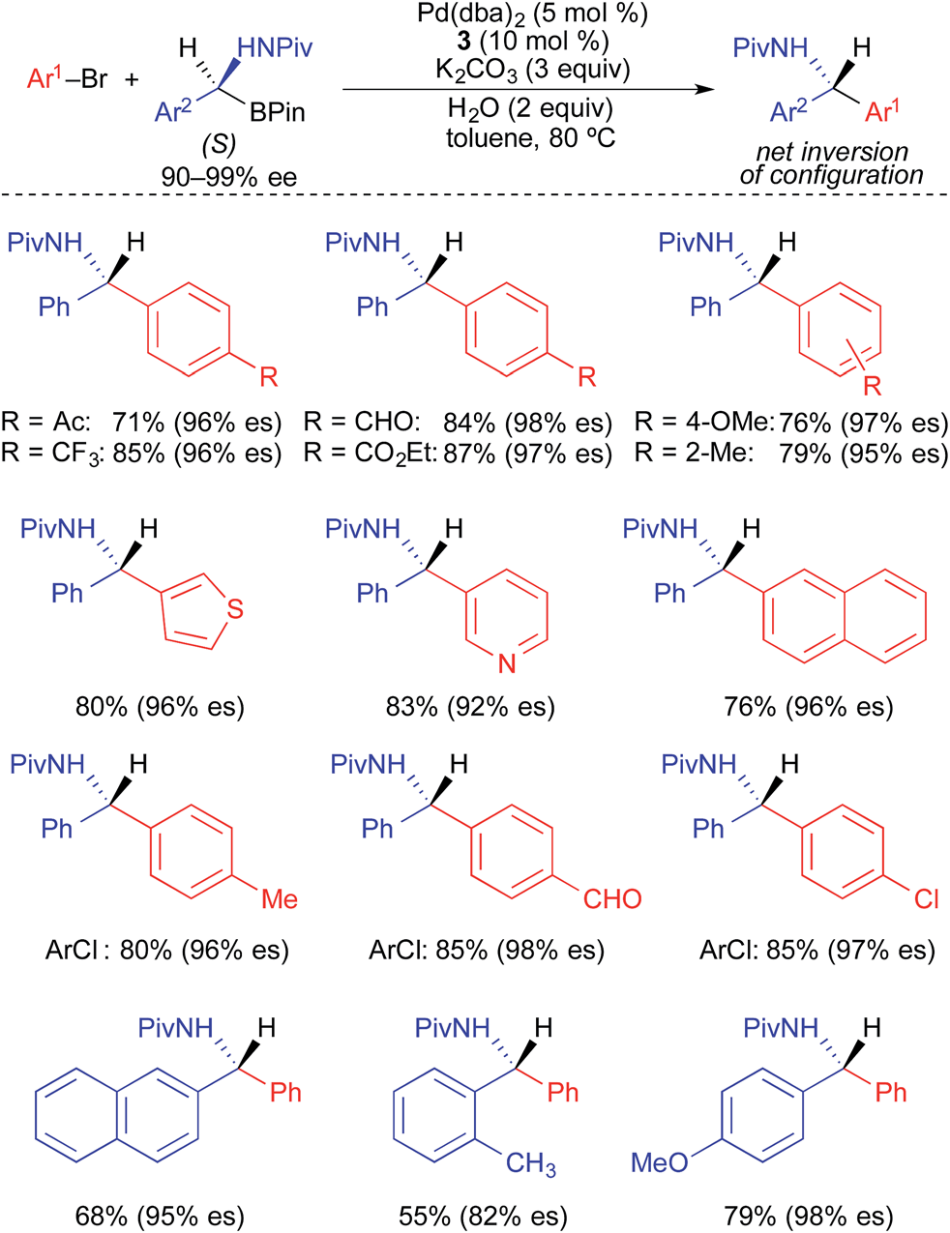
Stereospecific Pd-catalyzed cross-coupling reactions of enantioenriched α-(acylamino)benzylboronic esters and aryl electrophiles as reported by Suginome and Ohmura.

Suginome and Ohmura developed a stereospecific Pd-catalyzed process for the cross coupling of enantioenriched α-(acylamino)benzylboronic esters and aryl electrophiles using XPhos (**3**) as a supporting ligand (Fig. 13).^4*c*^ α-(Acylamino)benzylboronic esters bearing a pivaloyl-substituted amine were used broadly in highly stereospecific cross-coupling reactions with aryl bromides and aryl chlorides. The transformations proceeded with inversion of absolute configuration. Electron-rich, electron-neutral, and electron-deficient electrophiles, as well as *o*-substituted and heteroaryl electrophiles, were all well tolerated in these reactions. The use of α-(acylamino)benzylboronic esters bearing acyl groups smaller than pivaloyl (*e.g.*, acetyl and propionyl) resulted in significantly reduced enantiospecificity.

**Fig. 14:**
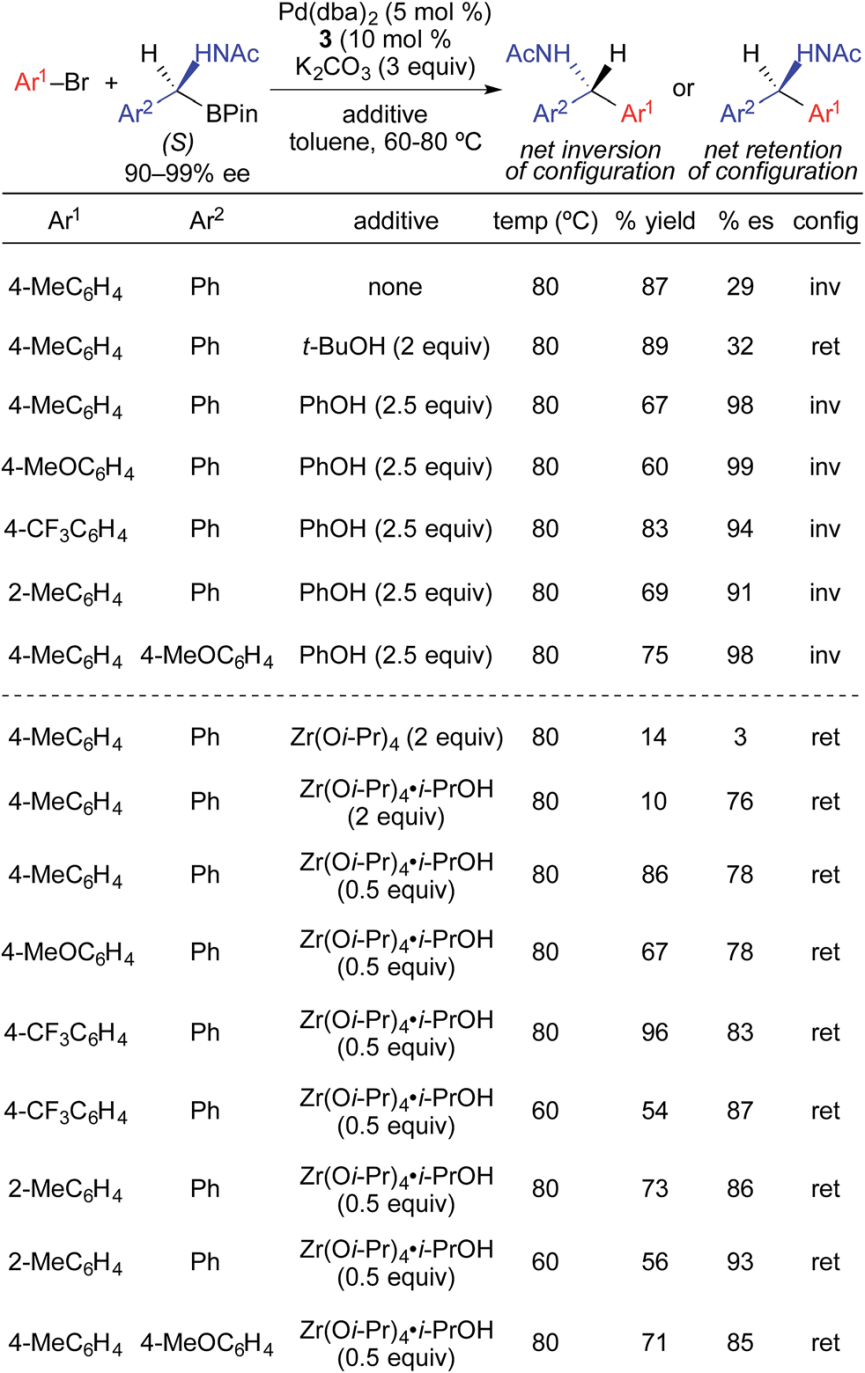
The effect of additives on the stereospecificity of Pd-catalyzed cross-coupling reactions of enantioenriched alkyl β-trifluoroboratoamides and aryl bromides as reported by Suginome and Ohmura.

Following their initial studies of stereospecific Pd-catalyzed cross-coupling reactions using α-(acylamino)benzylboronic esters, Suginome and Ohmura investigated the effects of protic and Lewis acidic additives on the enantiospecificity of the reactions (Fig. 14).^4*e*^ Transmetallation of α-(acetylamino)benzylboronic esters proceeded predominately through an enantioretentive pathway with *t*-BuOH, and predominately through an enantioinvertive pathway with PhOH. Using PhOH, highly enantiospecific cross-coupling reactions were achieved with electron-rich, electron-neutral, and electron-deficient aryl bromides. When phenol was replaced with metal Lewis acids, stereoretentive transmetallation was largely observed. The use of 0.5 mol% Zr(Oi-Pr)_4_·i-PrOH maximized reaction *via* the stereoretention pathway. As with the stereoinvertive reactions using phenol as an additive, the stereoretentive reactions using Zr(Oi-Pr)_4_·i-PrOH were successfully demonstrated for cross-coupling reactions using electron-rich, electron-neutral, and electron-deficient aryl bromides. Subtle changes in the reactions conditions resulted in dramatic changes in stereospecificity in these reactions. With Zr(Oi-Pr)_4_, the cross-coupling reaction occurred with only nominal stereoselectivity. Changing the acyl group from acetyl to pivaloyl, and the metal Lewis acid from Zr(Oi-Pr)_4_·i-PrOH to B(Oi-Pr)_3_, favored transmetallation *via* the enantioinvertive pathway.

**Fig. 15:**
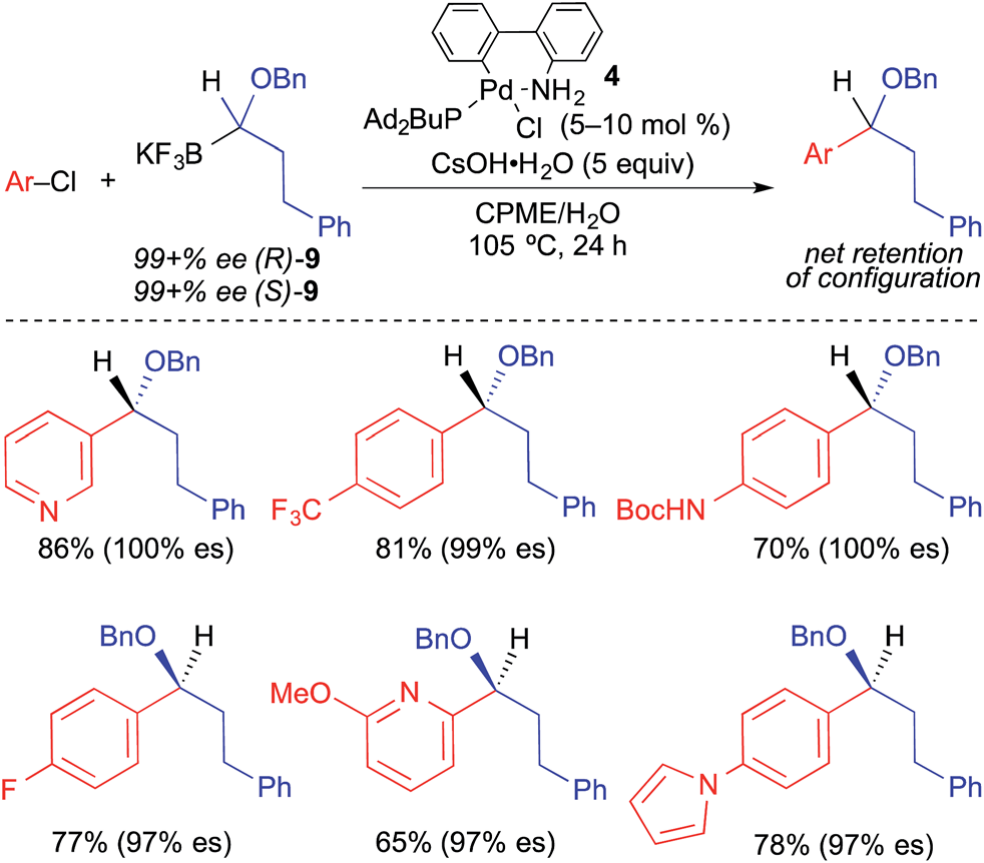
Stereospecific Pd-catalyzed cross-coupling reactions of enantioenriched 1-(benzyloxy)alkyltrifluoroborates and aryl chlorides as reported by Molander.

In 2012, Molander reported the stereospecific Suzuki cross-coupling reaction of 1-(benzyloxy)alkyltrifluoroborates and aryl electrophiles (Fig. 15).^4*g*^ Intramolecular coordination of the benzyl group to palladium was invoked as an essential structural element in this process. Second-generation Buchwald Pd precatalyst^22^ **4** bearing Ad_2_PBu promoted cross coupling broadly with aryl and heteroaryl chlorides. The enantiospecificity observed in this reaction was exceptionally high (97–100% es) and selective for inversion of absolute configuration. Notably, heteroaromatic chlorides were well tolerated by this process.

**Fig. 16:**
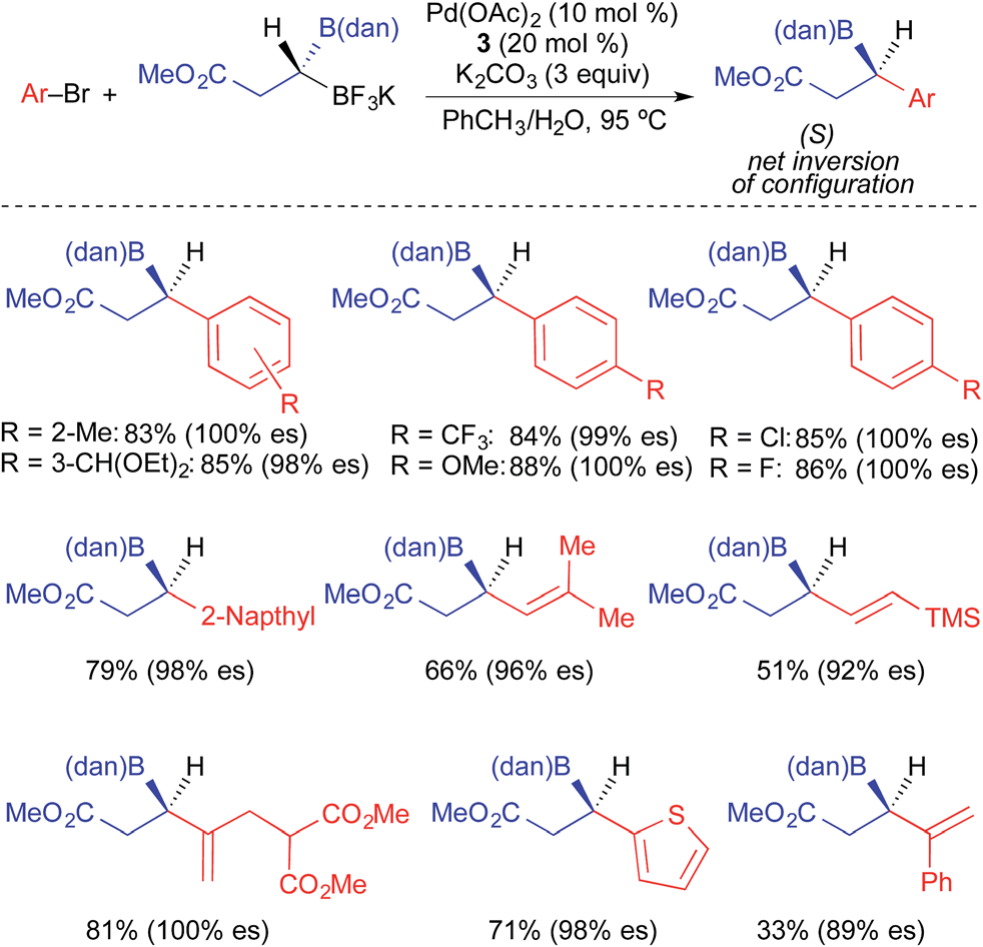
Stereospecific Pd-catalyzed cross-coupling reactions of enantioenriched 3,3-diboronyl carboxyesters and aryl/vinyl bromides as reported by Hall.

Hall reported the synthesis of enantioenriched 3,3-diboronyl carboxyesters, and their use in stereospecific Pd-catalyzed cross-coupling reactions with aryl bromides (Fig. 16).^4*f*^ The 3,3-diboronyl carboxyesters were comprised of a 1,8-diaminonaphthalenyl (dan) boron unit and a trifluoroborate unit, which enabled chemoselective and enantiospecific transmetallation at the stereogenic center when a catalytic system based on palladium and XPhos was employed. This reaction tolerated the use of electron-rich, electron-neutral, and electron-deficient aryl electrophiles, though only one example using a heteroaryl electrophile was provided. It was proposed that 3,3-diboronyl carboxyesters undergo facile transmetallation as a result of the cooperative effects of both carbonyl coordination to boron and stabilization imparted by the presence of an α-boronyl group on the Pd(ii) intermediate.^23^ Similar to prior results of Molander and Suginome, cross-coupling products were generated with inversion of absolute stereochemistry using β-carbonyl alkylboron nucleophiles.

**Fig. 17:**
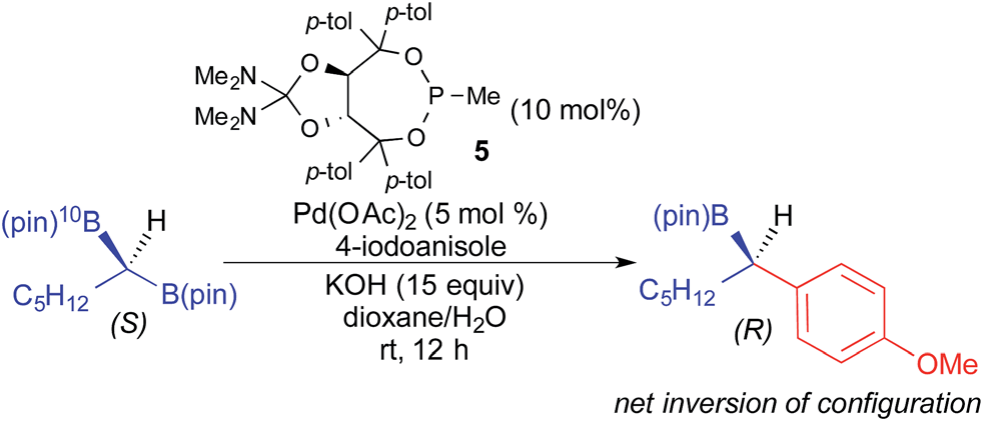
Stereoinvertive transmetallation in the Pd-catalyzed cross coupling of a geminal diboronyl reagent and an aryl iodide as reported by Morken.

In 2014, Morken reported the use of geminal diboronyl compounds in enantioselective Pd-catalyzed cross-coupling reactions using a chiral monodentate taddol-derived ligand (**5**).^4*i*^ Concurrently, Hall developed a similar process using taddol-derived phosphoramidite ligands.^4*m*^ In these reactions, achiral geminal bis(pinacolboronates) underwent enantioselective transmetallation and coupling to afford optically active organoboronic esters. Although these processes do not technically constitute stereospecific cross-coupling reactions, Morken demonstrated that transmetallation proceeds *via* a stereoinvertive mechanism through the elegant use of ^10^B labelling (Fig. 17).^4*i*^

**Fig. 18:**
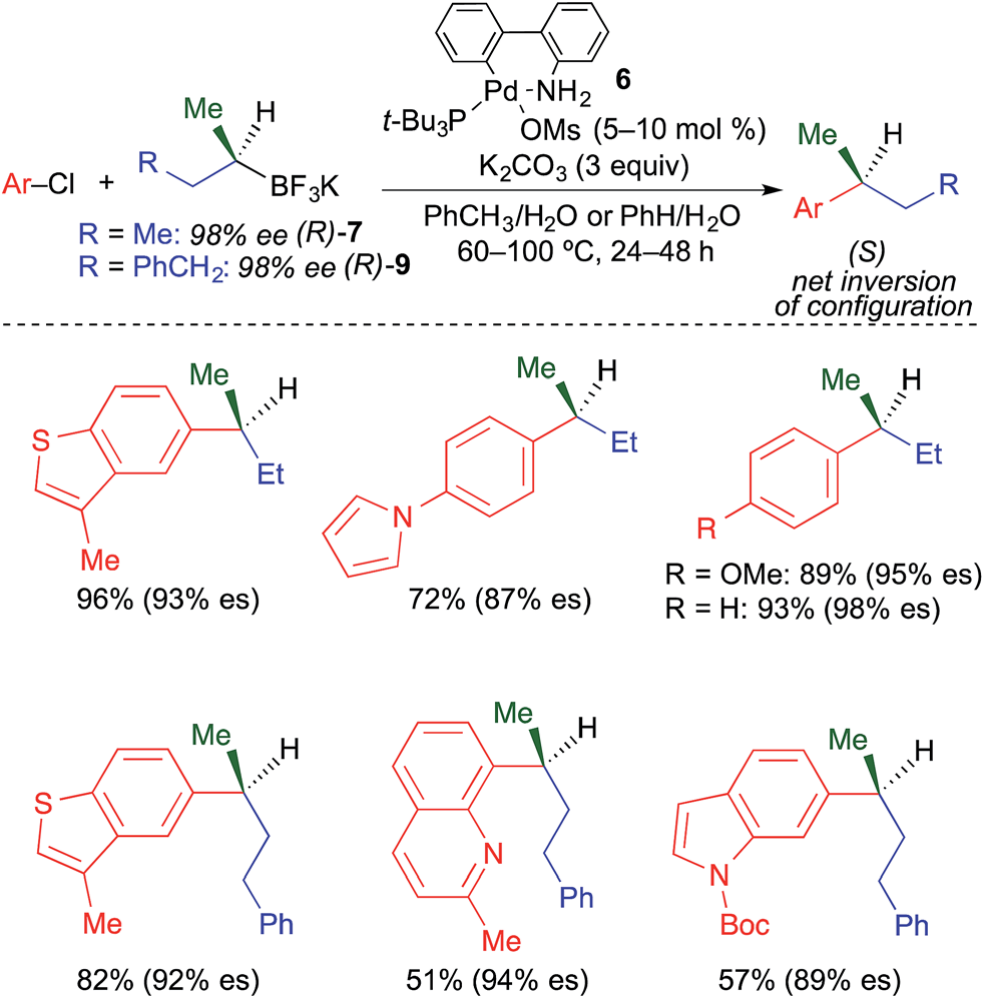
Stereospecific Pd-catalyzed cross-coupling reactions of unactivated, secondary alkyl trifluoroborates and aryl chlorides as reported by Biscoe.

Our group recently reported the first stereospecific Pd-catalyzed cross-coupling reaction of unactivated, enantioenriched secondary alkylboron nucleophiles and aryl electrophiles.^4*k*^ Using the combination of third generation Buchwald precatalyst^22^ **6** and K_2_CO_3_, the formation of isomerized cross-coupling products was fully inhibited. Unfunctionalized secondary nucleophiles such as s-BuBF_3_K were well tolerated in these reactions (Fig. 18). While the use of heteroaryl chlorides was demonstrated, heteroaryl chlorides with the chloride leaving group located directly on the heteroaryl ring (*e.g.*, 3-chloropyridine) were largely unreactive. Enantiospecificity in this process was generally high. Inversion of absolute configuration was observed in these reactions, which suggests that transmetallation of unactivated secondary alkyltrifluoroborates occurs preferentially through an invertive substitution mechanism.

## Mechanistic considerations

It is tempting to apply the previous precedents broadly to rationalize or predict the stereospecificity of new metal-catalyzed processes that employ optically active organometallic nucleophiles. However, these examples suggest that the stereoretentive S_E_2 and stereoinvertive S_E_2 pathways of transmetallation (Fig. 2) may be separated by small energy differences.^4e,24^ Thus, the observed stereospecificities are likely to be highly dependent upon multiple specific elements of individual cross-coupling reactions (*e.g.*, solvent, ligand, temperature, steric properties of nucleophile, presence of coordinating groups, nearby electronic perturbations, charge of active Pd complex). The work of Suginome, in which the addition of a Lewis acid reversed the pathway of transmetallation, highlights the sensitivity of individual reactions to subtle changes in reaction conditions.^4*e*^ Woerpel^25^ and Soderquist^26^ independently conducted seminal studies on the mechanism of transmetallation of primary alkyl-9-BBN nucleophiles. In each study, it was demonstrated that transmetallation occurs with retention of absolute configuration.^27^ In contrast, recent work from our group showed that transmetallation of unactivated secondary alkyltrifluoroborate nucleophiles occurs stereospecifically with inversion of configuration.^4*k*^ It is probable that imperfect enantiospecificity (% es) in Pd-catalyzed Stille and Suzuki cross-coupling reactions using enantioenriched nucleophiles arises from transmetallation *via* a minor secondary pathway. Such minor pathways may ultimately become dominant with appropriate modifications of reaction conditions, which would then result in formation of the opposite enantiomer. Careful, systematic studies of individual cross-coupling reactions will ultimately be necessary to deconvolute the factors that determine the mechanism of transmetallation in Pd-catalyzed reactions using alkyltin and alkylboron nucleophiles.

## Conclusion

Advances in Pd-catalyzed cross-coupling methods continue to facilitate the development of new, stereospecific cross-coupling reactions that employ configurationally stable, optically active, organometallic nucleophiles.^28^ In principle, such methods should serve as attractive alternatives to asymmetric catalysis for the generation of new stereogenic centers in non-racemic compounds. However, for stereospecific Pd-catalyzed cross coupling to reach its full potential as a general process for the manipulation of three dimensional structure, many challenges remain. Existing stereospecific transformations suffer from narrow substrate scopes, which severely limit their potential use. In most cases, the presence of a C(sp^2^) α-carbon, an α-heteroatom, and/or a coordinating group is necessary to facilitate transmetallation of a secondary alkyltin or secondary alkylboron reagent to palladium. The limited application of these methods to highly functionalized heterocyclic substrates underscores the need for stereospecific cross-coupling reactions with greater substrate scope. The potential for transmetallation to proceed *via* stereoretentive or stereoinvertive pathways additionally complicates the use of existing stereospecific cross-coupling reactions and the rational development of new stereospecific methods. Finally, the development of simple methods to prepare enantioenriched alkyltin and alkylboron reagents is required to encourage the wide adoption of the stereospecific approach. It is essential that these challenges be addressed if stereospecific Pd-catalyzed cross-coupling processes are to be developed and broadly implemented in asymmetric synthesis.
